# Emerging nanobiotechnology for precise theranostics of hepatocellular carcinoma

**DOI:** 10.1186/s12951-022-01615-2

**Published:** 2022-09-29

**Authors:** Mengjiao Xu, Liu Yang, Yanjie Lin, Yao Lu, Xiaoyue Bi, Tingting Jiang, Wen Deng, Lu Zhang, Wei Yi, Yao Xie, Minghui Li

**Affiliations:** 1 Department of Hepatology Division 2, Beijing Ditan Hospital, Capital Medical University, 8 Jingshun East Street, Chaoyang District, Beijing, 100015 China; 2 Department of Hepatology Division 2, Peking University Ditan Teaching Hospital, 8 Jingshun East Street, Chaoyang District, Beijing, 100015 China; 3 Department of Gynecology and Obstetrics, Beijing Ditan Hospital, Capital Medical University, 8 Jingshun East Street, Chaoyang District, Beijing, 100015 China

**Keywords:** Hepatocellular carcinoma, Tumor microenvironment, Nanobiomedical technology, Clinical theranostics

## Abstract

Primary liver cancer has become the second most fatal cancer in the world, and its five-year survival rate is only 10%. Most patients are in the middle and advanced stages at the time of diagnosis, losing the opportunity for radical treatment. Liver cancer is not sensitive to chemotherapy or radiotherapy. At present, conventional molecularly targeted drugs for liver cancer show some problems, such as short residence time, poor drug enrichment, and drug resistance. Therefore, developing new diagnosis and treatment methods to effectively improve the diagnosis, treatment, and long-term prognosis of liver cancer is urgent. As an emerging discipline, nanobiotechnology, based on safe, stable, and efficient nanomaterials, constructs highly targeted nanocarriers according to the unique characteristics of tumors and further derives a variety of efficient diagnosis and treatment methods based on this transport system, providing a new method for the accurate diagnosis and treatment of liver cancer. This paper aims to summarize the latest progress in this field according to existing research and the latest clinical diagnosis and treatment guidelines in hepatocellular carcinoma (HCC), as well as clarify the role, application limitations, and prospects of research on nanomaterials and the development and application of nanotechnology in the diagnosis and treatment of HCC.

## Background

Primary liver cancer mainly includes three different pathological types: hepatocellular carcinoma (HCC), intrahepatic cholangiocarcinoma (ICC), and mixed hepatocellular carcinoma cholangiocarcinoma (CHCC CCA). The three show great differences in pathogenesis, biological behavior, histopathology, treatment, and prognosis. HCC accounts for 75–85% of cases and ICC accounts for 10–15% of them. HCC is common in some Southeast African countries, including Southeast Asia, the Western Pacific, and Sub-Saharan Africa. The incidence rate of liver cancer in 50% of regions is over 30/100 thousand. Australia, Europe, and the United States are low incidence areas, with an incidence rate of less than 5/100 thousand. The incidence rate grows with each passing day in many countries [1–3]. HCC has become the main cause of cancer and cancer deaths in China. In high hepatitis B virus (HBV) and hepatitis C virus (HCV) infection areas, hepatic cirrhosis has become a serious problem. The incidence rate of liver cancer increases by 7.9% annually. Studies have shown that hepatocellular carcinoma has the highest incidence rate of primary liver cancer. Risk factors for liver cancer include liver fibrosis and inflammatory cirrhosis, aflatoxin-induced toxicity, smoking, hereditary hemochromatosis, metabolic disorders such as diabetes and non-alcoholic fatty liver disease, and immune-related diseases including primary biliary cirrhosis and autoimmune hepatitis [4, 5].

Early hepatocellular carcinoma can be treated by a variety of means, including surgical resection, radiofrequency ablation, absolute ethanol injection, and chemoembolization. Surgical treatment is the most important means for liver cancer patients to obtain long-term survival, mainly including hepatectomy and liver transplantation. The Guidelines for the Diagnosis and Treatment of Primary Liver Cancer (2022 Edition) [6] recommend surgical resection as the first choice for HCC in stages Ia, Ib, and IIa of the China liver cancer (CNLC) staging system with a good liver reserve function. However, these patients only account for about 20% of the total, and the 5-year survival rate is not ideal, with over 70% of patients relapsing within 5 years [7–9]. Majority of the HCCs is diagnosed at advanced stage when the optimal time for curative treatment is missed. Palliative systematic treatment is the only option for most patients with advanced-stage HCC [10–12]. Chemotherapy drugs for HCC are mainly divided into targeted therapeutic drugs and immunotherapeutic agents. There are few first-line therapeutic drugs, with limited options, a low objective remission rate, and limited survival benefits. After first-line treatment failure, the second-line treatment is stretched, and drug accessibility is poor, making the HCC cure rate low [13–20].

**Fig. 1:**
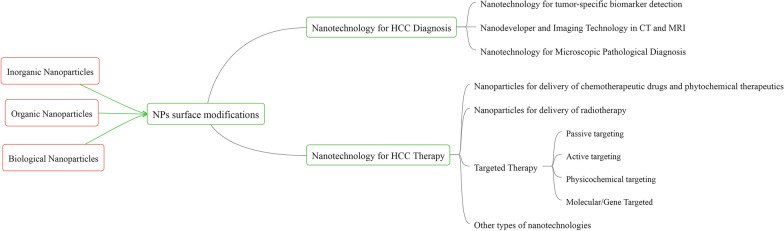
Mind map of the manuscript

At present, the monitoring and diagnosis of liver cancer mainly rely on imaging examination and blood tumor markers. Most HCC cases [21] were found in a late clinical stage, the tumor progressed rapidly, and there were many adverse reactions to treatment. Therefore, current diagnosis and treatment methods could not fully meet the clinical needs, and there is an urgent need for new scientific and technological means to comprehensively diagnose and treat HCC in clinical practice [22]. The principles and methods of nanoscience and nanotechnology have been combined with medicine, leading to more sensitive and faster nanomedical diagnosis technologies and more effective treatment methods, such as nano high-performance computed tomography (nano-CT), nano-magnetic resonance imaging (nano-MRI), nano-fluorescence imaging and other efficient molecular imaging modes, as well as new diagnosis and treatment integration strategies including photodynamic, acoustic dynamics (Fig. 1), laser ablation, and magnetothermal ablation. At the same time, nanotechnology also enables us to understand the process and mechanism of life activities at a more micro level [23–26].

## Surface engineering of nanoparticles and its interaction mechanism with liver

### Nanoparticles (NPs)

Nanoparticles (NPs) have a large specific surface area with high stability and have light, sound, electricity, and magnetism properties, which make them more suitable for drug absorption and subsequent controlled-release utilization. Because the structural integrity of tumor blood vessels is poor and the gap between tumor blood vessels is largerer than that of normal blood vessels, nanoparticles can be beneficial for targeting tumors. Compared to the traditional treatment modes, nanoparticles have broader application prospects [27, 28]. Compared with low molecular weight drugs, nanodrugs can protect the payload before delivery [29]; have higher drug loading capacities, targeting specificity, and accuracy; and show more opportunities for sustained release due to their multivalent state. This allows the adequate absorption of epithelial tissue and prevents the degradation of binding drugs, changing the pharmacokinetics and distribution characteristics of drugs, improving the optimal concentration of anticancer drugs in cells in a short time, reducing the side effects of cytotoxic drugs, and improving the curative effect of therapeutic drugs, thus rendering them excellent tumor-targeting carriers [30, 31]. Generally, NPs can better penetrate the vascular wall and remain in tumor tissue with sized 10 ~ 200 nm, in a process called enhanced permeability and retention (EPR effect) of tumor blood vessels. However, transitional research scale of "nano" materials made it difficult to strictly definite the scope limit [32, 33]. EPR is the premise that nanodrugs can be selectively distributed in tumor tissues, having good targeting and low toxicity and side effects [34–36]. Therefore, this drug delivery system (DDS), which selectively delivers therapeutic drugs to tumor sites, is gradually showing great potential in the targeted treatment of HCC.

### Liver entry mechanism of nanomaterials

Although nanoparticles have better tumor enrichment characteristics than traditional drugs, the barrier effect of the liver on foreign substances is a major issue. Research shows that most nanodrugs deposited by oral or intravenous injection will be found in the liver and spleen and metabolized out of the body, resulting in reduced bioavailability, far lower treatment effects than expected, and even liver toxicity and a series of metabolic problems. The structural basis of nanoparticle uptake by the liver is the reticuloendothelial phagocytosis system (RES) [37], composed of Kupffer cells. Nanoparticles entering the blood circulation will first bind to plasma proteins, which will be rapidly opsonized and further mediate the endocytosis of macrophages, so that they can be deposited in the liver and be prevented from entering the blood circulation again.

Additionally, after observing the uptake of nanoparticles by the liver in real time through multiphoton fluorescence microscope, the scavenging effect of the liver was found to be related to the hydrophobicity, surface charge, and size of nanoparticles [38]. Negatively charged particles are more likely to be uptaken by the RES, resulting in liver toxicity, while hydrophilic groups are conducive to escape from the barrier effect of the liver. Research [39] has shown that nanoparticles with a diameter of 60 nm are the most conducive to enrichment in tumors. Increases or decreases in diameter can increase the deposition in the RES to a certain extent. Previous studies [40] have deeply analyzed the barrier capacity of NPs in liver from the perspective of cellular phenotype, tissue structure, and hemodynamics. Nanoparticles are more likely to accumulate in the vascular plexus. The rich blood supply and large blood flow exchange area in the hepatic sinusoid lead to slow blood flow, which is an important reason for the easy deposition of nanoparticles in the liver. Additionally, the phenotype of macrophages in the liver and spleen also plays a decisive role in nanoparticle uptake. Macrophages differentiate into Kupffer cells and red pulp cells in the liver and spleen, respectively. Compared with ordinary macrophages, their phagocytosis is significantly enhanced.

### Functional modification of nanoparticles

To achieve the escape of RES, researchers modified the targeting ligand on the surface of nanoparticles to improve the ability of drug carriers to actively target cells, weaken the liver barrier, and reduce the toxic and side effects of drugs, so as to effectively release drugs to specific cells and achieve targeted drug delivery. Therefore, the multi-functional modification of nanocarriers has become a research hotspot in recent years. Polyethylene glycol (PEG) is the most common modification strategy as it can keep nanomaterials from combining with plasma proteins, block the opsonization process, and escape the phagocytosis by the RES [41]. Additionally, PEG can prolong the blood circulation time and improve biocompatibility, which has become a recognized effective improvement method. Due to the overexpression of folate (FA) receptors on the surface of most liver cancer cells, Chi et al*.* [42]constructed nanoparticles ((M-LPMSN)-NiAsOx) loaded with a macroporous silica ATO prodrug (NiAsOx). FA improved the targeting ability of nanoparticles and endocytosis, thus increasing the absorption of nanoparticles by tumor cells, showing stronger antitumor effects than MPEG modification. At present, the surface of nanomaterials can also be modified with peptides, small molecules, antibodies, vitamins, and other biological elements [43, 44]. Studies have shown that the characteristics of vascular leakage, the microenvironment, and surface receptors of hepatocellular carcinoma are most related to the functional design of nanoparticles. In addition, it can also be artificially modified according to the characteristics of acidic pH value of tumor area [45, 46], high glutathione content [47–49], high osmotic pressure of tumor stroma [50–52], and hypoxia between tissues [53]. Agents used for the diagnosis and therapy of liver cancer are listed in Tables 1 and 2.

**Table 1:** Diagnostic Nanocarriers Delivery Agents for Hepatocellular Carcinoma

Nanocarriers	Diagnostic Agent	Technique	Target cell	References
Nanobubbles (NBs)	Nanobubbles; Pro-bexarotene NBs; NBs-GPC3-rGO; Poly(oxalate-co-curcumin) (POC)	Ultrasound imaging; Nano-knife	HCC cell lines, HepG2	[54, 55]
Dendrimer	Polydopamine	CT and PTT	HepG2	[56, 57]
Carbon Dots (CDs)	Gd	MRI	HepG2	[58]
Calcium phosphate NPs	Gd-DTPA	MRI	BEL-7402,HepG2	[59]
Silica NPs	Gd; Gold NPs	MRI and PDT	BNL 1 ME A. 7R.1 cell	[60, 61]
Graphene Oxide-Based Nanocarriers	Gd	MRI	Human hepatoma cells	[62–64]
Metal oxide NPs (Iron, Zinc)	Gd	MRI	HepG2	[65, 66]
Superparamagnetic Iron-Oxide NPs(SPIONs)	Gd; Iron oxide	MRI	HCC cell lines	[67–69]
Micelles	Micelles; SPION; Gd	MRI/UCL	HepG2	[70, 71]
Lipid NPs; Vesicle NPs; Liposome	Gd; Quantum dots	MRI/PAI	HepG2	[72–74]
Polymeric NPs	Gd-DTPA; SPION	MRI	HCC cell lines	[75, 76]

**Table 2:** Therapeutic Nanocarriers Delivery Agents for Hepatocellular Carcinoma

Nanocarriers		Drugs	Target cell	Anticancer Mechanism	Outcomes
Inorganic	Nanoshells	Sorafenib; Doxorubicin; Oxaliplatin	HepG2	Photothermal therapy; Codelivery of drugs	Inhibit cell proliferation and minimize the resistance of HCC
	Inorgnic Nanofibers	Doxorubicin	SMMC-7721, H22 tumor	Delivery of drugs	Provide good tumor-targeting activity; Prolonged drug release with lower tumor cytotoxicity; Prevent tumor recurrence
	Graphene Oxide-Based Nanocarriers	Doxorubicin	Hepatoma cells	Delivery of drugs	Exhibit a cytotoxic effect on liver cancer cells
	Calcium NPs	Sorafenib; Doxorubicin; Immunosuppressive agents; Therapeutic genes	HepG2, HepG2/ADR,SMMC-7721,A549,BEL-7402	Delivery of drugs and genes	Enhance anticancer effect with lower cytotoxicity
	Metal oxide NPs (Iron, Zinc, Alumina)	Sorafenib; Doxorubicin; Paclitaxel; Therapeutic genes	Hep-2,HepG2,Hep3B,Hepa1-6,Huh-7,SMMC-7721	Membrane disruption/ROS production; Delivery of drugs and genes; Magnetic field induces lysosomal leakage; Magnetic hyperthermia	Induce cytotoxicity and apoptosis of HCC cells; Efficient tumor imaging
	Silver NPs	–	HT29,HepG2	ROS formation/apoptosis induction; Disruption of intercellular proteins; Apoptosis through caspase expression	Induce apoptosis of HepG2 cells
	Platinum NPs	–	HepG2	Release of Pt(II) ions	Enhance antitumor activity with fewer side effects
	Gold NPs	Cisplatin; Doxorubicin; Capecitabine; 5-FU; Therapeutic genes	HepG2 & resistant HepG2, HepG2-C3A, H22, HCC-LM3-fLuc, Huh-7, HepB3, HepB5, A549, MCF-7	Cationic cellular uptake/oxidative stress; Cell uptake through peptide conjugation; Gold atom-intercellular protein interaction; *c-Myc* gene silencing by siRNA; Delivery of 5-FU; photothermal therapy; Immunotherapy, conjugation with SM5-1; Gold-protein interaction/Radiotherapy; miR-375 replacement therapy	Effectively inhibit the growth and resistance of HCC cells; Reduce toxicity systematically
	Silica NPs	Doxorubicin; Cetuximab; Cisplatin; Therapeutic genes	H22SMMC-7721 cell lines, HepG2, PLC, BEL-7402, QGY7703	Delivery of drugs; Suicide gene delivery and gene regulation; Magnetic hyperthermia; Photothermal therapy	Effective drug-releasing ability and efficient tumor-homing; Better antitumor activity; Reduce toxicity systematically
	Selenium NPs	Doxorubicin; Baicalin Therapeutic genes	HegG2,HepG2215	Delivery of drugs and genes	Inhibit tumor growth; Mitigate harmful effects from almost any drug at the delivery area
	Janus NPs	Doxorubicin; Docetaxel	HepG2,H22	Delivery of drugs; Photothermal therapy	Effectively enhance tumor targeting and internalization in selective and safe chemotherapy for multidimensional HCC theranostics
	Magnetic Nanoclusters	Doxorubicin	HCC cell lines	Sustained drug loading and release; In vivo MRI of intra-tumoral delivery	Significantly enhance liver cancer cell death
	Superparamagnetic Iron-Oxide NPs (SPIONs)	Doxorubicin; Curcumin	HCC cell lines,HepG2	Delivery of drugs and as therapeutics themselves; Targeted MR imaging of HCC; Magnetic hyperthermia	Effectively inhibit HCC cell growth
Organic	Chitin and chitosan-based NPs	Doxorubicin	HepG2,H22,LO2,HU7,SMMC-7721	Delivery of drugs; mRNA apoptotic gene expression	Kill HCC cell lines; Higher antitumor activity; Stronger fluorescent intensity shown in tumor tissue
	Polysaccharide NPs	Doxorubicin; Paclitaxel	HepG2,H22,SMMC-7721	Delivery of drugs	Intrinsic liver targeting capability to incorporate multiple targeting and therapeutic approaches
	Micelles	Sorafenib Doxorubicin	HCC cell lines, HepG2	Provides specific targeted action against HCC	Good tumor growth inhibition and overall survival rate
	Albumin NPs; Peptide NPs	Doxorubicin; Paclitaxel; Therapeutic genes	HepG2,Huh7	Delivery of drugs and genes	Significantly enhance antitumor efficiency in HCC
	Other polymer NPs	Doxorubicin; 10-hydroxycamptothecin; Therapeutic genes	HepG2,Hep3B,Hepa-1.6,C3A,SK-HEP-1	Delivery of drugs and genes; Photothermal therapy	Multi-functionality of DDS in specific antitumor therapy
	Organic Nanofibers	Cisplatin; Doxorubicin; Therapeutic genes	HCC cell lines,EMT6	Delivery of drugs and genes	Provide good tumor-targeting activity; Significantly inhibit metastasis and tumor growth of liver cancer cells; Prevent tumor recurrence
	Carbon Nanotubes	Doxorubicin	HCC cell lines,SMMC-7721,H22	Delivery of drugs; Specific cancer imaging and selectivity of HCC; Photothermal therapy	Kill HCC cell lines; Repress liver cancer growth
	Carbon Dots (CDs)	Doxorubicin	HepG2,HL-7702	Delivery of drugs; Active anticancer effects through direct pyrolysis of an organic therapeutic; Restrains migration for adjuvant therapy; Increased efficiency of radiotherapy	Multi-functionality of DDS in specific antitumor therapy
	Liposomes	Cisplatin; Sorafenib; Doxorubicin; 5-FU; Therapeutic genes	HepG2,H22	Delivery of drugs and genes	Longer imaging time and higher signal enhancement; Good inhibition of cell growth
	Lipid NPs; Vesicle NPs	Sorafenib; Doxorubicin; Therapeutic genes	HepG2, Hep3B, Huh7	Delivery of drugs and genes	Significantly enhanced antitumor effects Efficiency in HCC
	Hydrogel NPs	Cisplatin; Therapeutic genes	HepG2, Hep3B, H22	Delivery of drugs and genes	Efficient intracellular drug release and subsequent induction of tumor cell death
	Dendrimer	Doxorubicin	HepG2	Enhance the uptake of drug; Increases the cytotoxicity and anticancer effect	Better antitumor efficiency
	Polyethylene glycol (PEG) NPs	Sorafenib; Vandetanib	HepG2, BEL7402	Delivery of drugs	Capability of producing promising drug delivery
	Polylactic-Co-Glycolic Acid (PLGA) NPs	Sorafenib; Selumetinib; Docetaxel Brucine; Therapeutic genes	HepG2,Hep3B, HCC1, SK-Hep1, JHH-7,BEL7402,SK-Hep1,HL-7702	Delivery of and genes	Increase drug loading capacity and biocompatibility; Effectively suppress tumor cell proliferation; Improve antitumor efficiency
	Metal–organic frameworks (MOF) NPs	Dihydroartemisinin	HepG2,HCT116,MCF-7	Delivery of drugs	Enhanced antitumor efficiency in HCC with low cytotoxicity and side effects
	Upconversion NPs	doxorubicin and hydroxycamptothecin(HCPT)	HepG2	Phothermal therapy; codelivery of drugs	Multimodal antitumor function in reducing tumor volume to almost zero in vivo

As the main organ of nanopreparation deposition, the liver is the main barrier restricting the role of nanodrugs. Research on the mechanism of liver nanoparticle uptake facilitates the improvement of existing technology. Thus, the regulation of the corresponding structure and function of nanopreparations can be constantly improved, allowing the design of nanocarriers with stronger specificity for the characteristics of the liver cancer microenvironment, and broadening the application prospects of nanomedicine in liver cancer diagnosis and treatment.

## Research status of nanocarriers in liver cancer diagnosis

Ultrasound, CT, and MRI are the main clinical methods for the diagnosis of liver cancer. The rise of nanomedicine has greatly changed the traditional imaging mode and promoted the development of molecular imaging. The latter refers to the display of changes at the molecular level in the living state and the early diagnosis of diseases at the tissue and cell levels. Based on the following characteristics, nanoparticles can be used as molecular imaging probes in vivo. Firstly, the particles are small, particle size distribution is narrow, and particles have a large specific surface area. Secondly, medical nanomaterials with good targeting, excellent imaging properties, and good biocompatibility can be obtained. Additionally, compared with traditional fluorescence methods, this method can provide a more complete vision of tumor resection, which is less costly and can improve tumor prognosis.

### Nano diagnostic materials for liver cancer

#### Inorganic nanoparticles

Inorganic nanoparticles include quantum dots, iron oxide nanoparticles, gold nanoparticles, and nanodiamonds. Current research on them mainly focuses on the construction of a drug release system integrating chemotherapy, targeting, and magnetic resonance imaging (MRI) with mesoporous silica, gold nanoparticles, and sodium polyacrylate inorganic nanoparticles as drug carriers and the tumor microenvironment as the main stimulation response mechanism [77]. Shi et al.[78], considering the fact that traditional nanodrug delivery systems cannot recognize the nuclear pore complex or effectively cross the nuclear pore (20–70 nm), designed and constructed an ultra-small mesoporous silica (25 nm) nanodrug delivery system with a nuclear targeting function. In the cytoplasm, the drug loading system can intelligently identify the nuclear pore complex and avoid its exclusion of heterostructures, so as to cross the nuclear membrane and enter the nucleus, releasing the drug in situ around the nuclear target to significantly improve the DNA damage effect of anticancer drugs, greatly enhance the anticancer effect, and provide a new drug delivery method for the clinical realization of high-efficiency and low-toxicity chemotherapy effects.

#### Organic nanoparticles

Organic nanoparticles are important nanomaterials. The first FDA-approved nanodrug was based on nanocarrier liposomes. Most carbon-based, NPs are in this section, others such as dendrimers, emulsions, aptamers, solid lipid NPs, nanobodies, and other polymers are considered organic particles. PEG-PLGA copolymers have been frequently used for HCC treatment, as both are commonly used and FDA-approved for different applications [79–81]. Nowadays, the most used metal organic framework materials have potential applications in many fields, because of their unique high porosity, structural characteristics, adjustable pore size and functionalization. Living cell research shows that metal organic framework materials coated with proteins can be quickly ingested by cells, and the proteins can be effectively released from the endolysosome of living cells and escape to the cytoplasm while maintaining the natural activity of the protein [82–84]. Wang et al*.* [85] bonded ultra-small gold nanoparticles (Au NPs) with a glucose oxidase-like effect on a metal organic framework (MOF), loaded with the chemotherapy drug camptothecin (CPT) and modified with C_12_SH and PEG-SH to improve stability, and used the glucose oxidase-like effect combined with a Fenton reaction to promote drug release and achieve a chemokinetic therapy cascade. This effectively solved the problem of insufficient endogenous H_2_O_2_ in tumor cells faced by chemodynamic therapy, and provided a new idea for the design of advanced nanodrugs for chemotherapy/chemodynamic synergistic therapy. Upconversion NPs can also generate a multifunctional DDS for liver cancer treatment through the co-delivery of hydroxycamptothecin and doxorubicin [86].

#### Biological nanoparticles

**Fig. 2:**
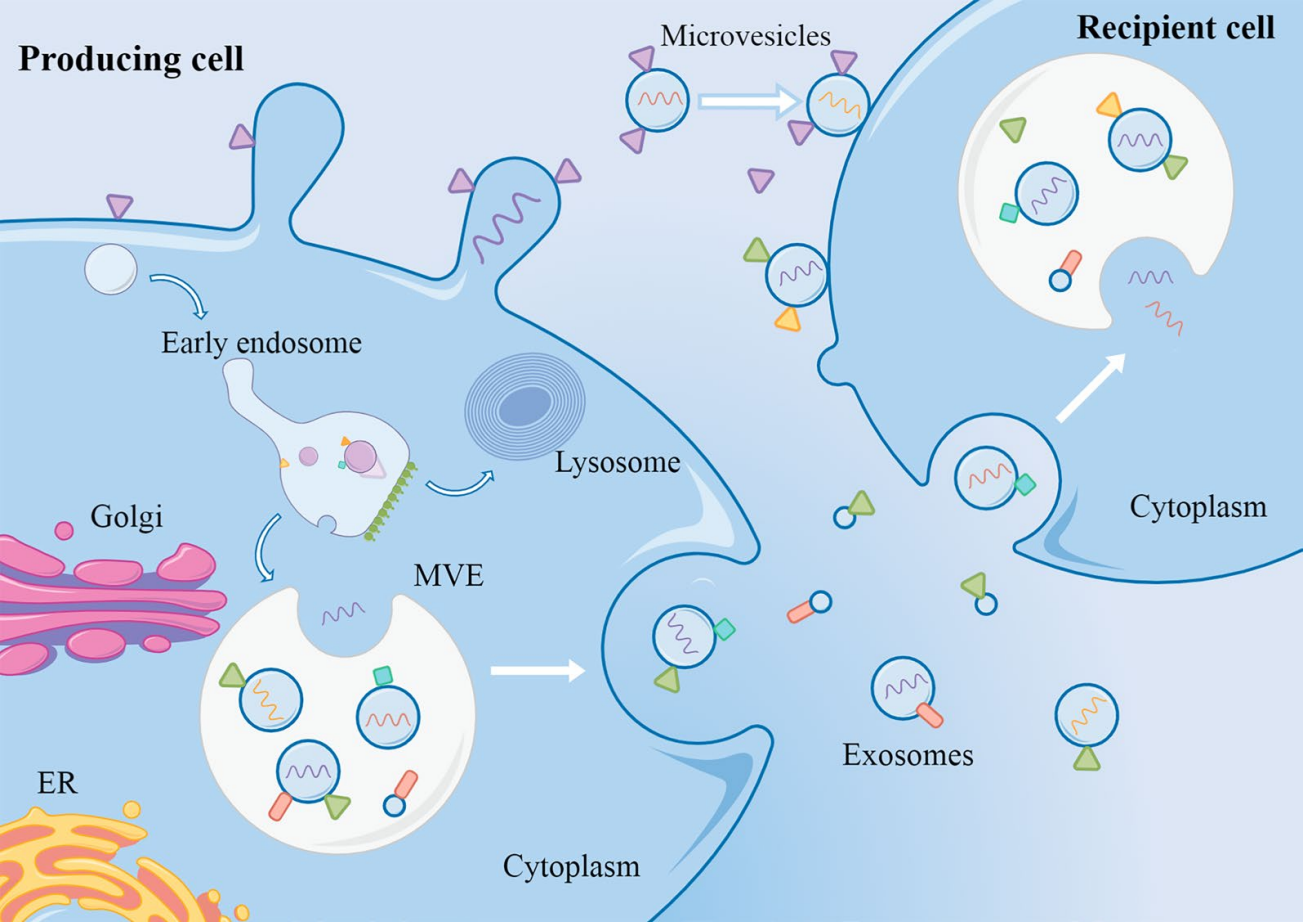
Cell–cell communication by extracellular vesicles

Extracellular vesicles (EVs) are a group of heterogeneous lipid-binding nanoparticles, including exosomes and microcapsules, which are considered to be the central medium of intercellular communication [87–90]. EVs target receptor cells by specifically binding ligands, and transfer their surface or intraluminal contents to the cytoplasm to exert their specific functions (Fig. 2, By Figdraw, www.figdraw.com). Accumulating evidence shows [91–94] that cancer-derived EVs can promote the differentiation of cancer-related cells, enhance angiogenesis, and regulate innate and adaptive immune responses, thus affecting tumor progression. In recent years, EVs have being used in accurately control the size and polydispersity of NPs by through microfluidic flow elements, reduce the immune clearance rate of delivered drugs, and play an important role in the optimal drug loading design of DDS.

Nanomaterials can not only carry drugs but also be used for RNA interference therapy of liver cancer. Non-viral nanoparticles can combine with the receptor on the cell surface to produce biofilm fusion, and then safely deliver various forms of molecular biological products such as DNAs, siRNAs, proteins, or polypeptides to the target cell to prevent it from being degraded by nucleases [95, 96]. Cytonacx developed by RNtein Biotechnology is a biological nanoparticle with a shell membrane structure. Nowadays, a variety of Cytonacx-carrying special DNAs have been described, affecting mRNA expression and causing corresponding protein deletion to induce apoptosis and inhibit tumor cell proliferation. After PEG surface modification, Cytonacx has the function of continuous and efficient molecular biological transformation of cells and overcomes the side effects, easy mutation, and toxic immune reactions caused by recombinant viral vectors in gene therapy. More strikingly, it can be assembled in a cell-free environment to achieve high-quality and large-scale preparations [97].

### Research and development of nanodiagnostic technology for liver cancer

#### Diagnostic technology based on molecular markers

Tumor markers are important indicators for the clinical auxiliary diagnosis of tumors, therapy indications, and prognosis evaluation. Compared with other tumors, liver cancer cells will typically express a variety of membrane protein molecules [98, 99]. These characteristic proteins are often used in the early screening of liver cancer. In nanomedicine, this characteristic is also key for the surface modification and functional design of nanocarriers.

Molecular markers (antigens, antibodies, enzymes, hormones, etc.) can be recognized by electrochemical biosensors, and be applied to fluorescence immunoassay technology. By transforming the signal of target molecules and their reaction into electrical signals, the qualitative or quantitative detection of biological macromolecules can be realized. Two DNA strands with specific aptamers and base complementary sequences are used as probes. At the same time, a target protein is identified to trigger the “proximity binding” of the nucleic acid probe and the DNA strand replacement reaction. The released single-stranded DNA is recognized by nanochannels and output signals, achieving the quantitative analysis of the target protein [100].

Liang et al *.*[101] developed a sandwich immunosensor labeled with double-layer enzyme-modified carbon nanotubes. The electrochemical biosensors of carcinoembryonic antigen and alpha-fetoprotein were constructed by double electrical signal amplification and specific molecular imprinting, which provided strong support for the hypersensitive detection of clinical parameters. Through the transformation of PdPt nanoparticles, Liu et al.[102]*.*achieved a sensitive and simultaneous determination of a variety of tumor markers, demonstrating that immunosensors constructed of nanoalloy materials can provide richer active sites of catalytic reaction and have better biocompatibility and synergy. The frequency shift immunoassay method, based on multiple surface-enhanced Raman scattering (SERS) developed by Tang et al*. *[103], can improve the detection sensitivity to the order of 10 − 13 m, which can be used to accurately detect the serum marker glypican-3 (GPC-3) and alpha-fetoprotein (AFP). Other researchers [104] designed a new label-free chemiluminescence (CL) immunoassay for the determination of AFP by using CuS nanoparticles as peroxidase simulants. Compared with the CL immunoassay based on enzyme labeling, the proposed label-free assay is simpler, cheaper, and faster. The linear range of using label-free CL immunoassay for the determination of alpha-fetoprotein is 0.1 ~ 60 ng/ml, and the detection limit is 0.07 ng/ml, showing good specificity and acceptable repeatability and accuracy.

**Fig. 3:**
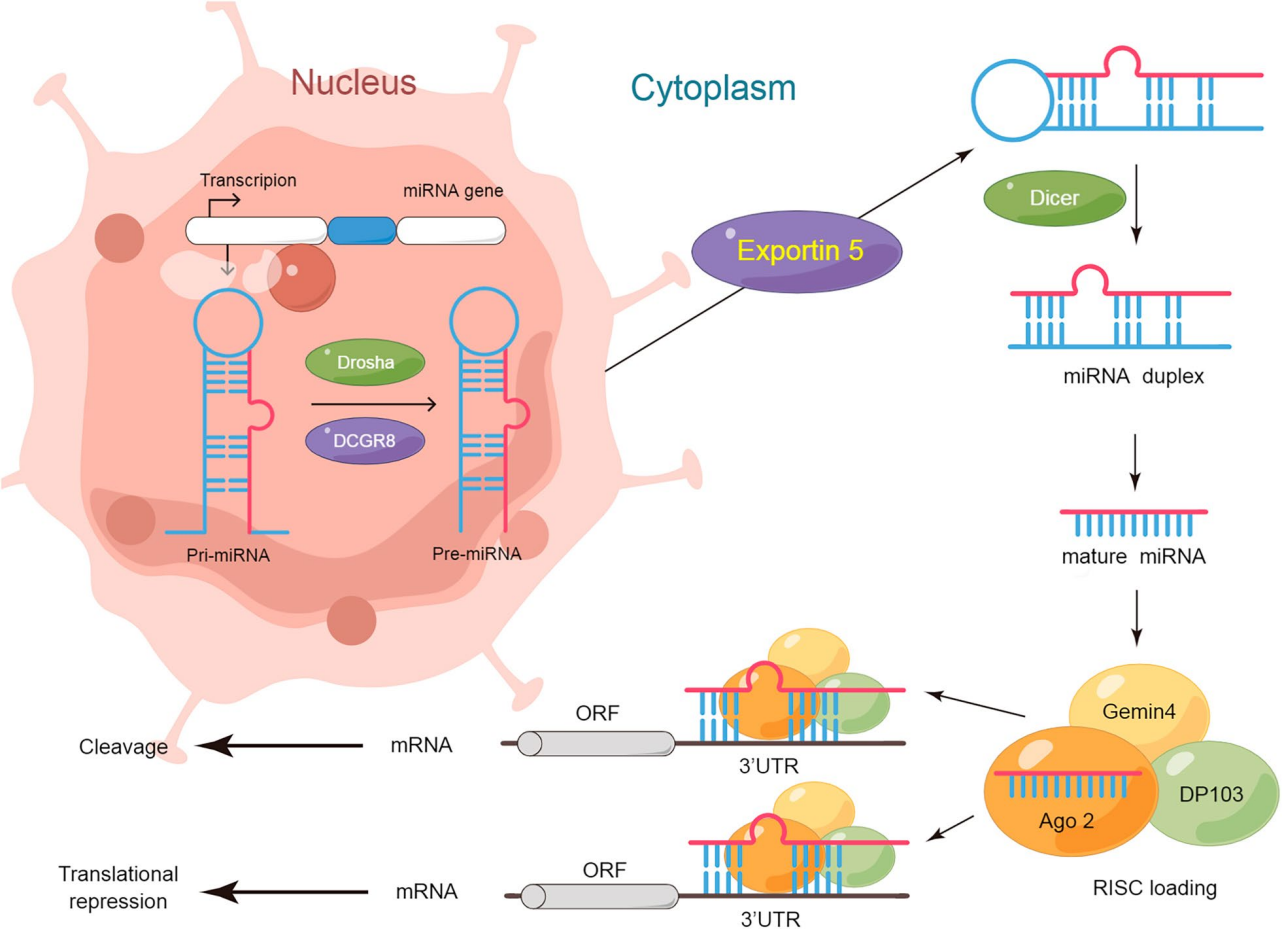
Representation of the production and mechanism of miRNAs

Micro RNAs (miRNAs) are single-stranded small-molecule (around 21–23 bases) RNAs. They are produced from the single-stranded RNA precursors (around 70–90 bases) with a hairpin structure, after being processed by the enzyme Dicer. Their mechanism of action is shown in Fig. 3 (By Figdraw, www.figdraw.com). The levels of miR-122, miR-21, and miR-233 in the plasma of patients with hepatocellular carcinoma were significantly different from those of patients with liver cirrhosis and hepatitis and healthy individuals [105]. miRNA7 ™ Technology [106] has higher sensitivity than that of AFP above 30%, a commonly used tumor marker of liver cancer, and its specificity is up to 90%. It can not only make up for the lack of negative AFP detection in 40% of HCC patients but also greatly improve the detection rate of early and very early liver cancer.

Famous scholars from America [107] and Japan [108] have confirmed that peripheral blood EpCAM + CTC is the “seed” of liver cancer metastasis and recurrence and can be used as an independent predictor of metastasis and recurrence after liver cancer resection. It is efficient in the early warning of liver cancer metastasis and recurrence, achieving it 4.8 months earlier than imaging and 8.5 months earlier than AFP. The liver cancer miRNA detection kit and CTC sorting detection system can carry out risk early warning and the dynamic monitoring of curative effects for HCC patients. They have been included in the code for diagnosis and treatment of primary liver cancer of the National Health Commission in 2019, to achieve the early diagnosis and treatment of HCC patients and improve their overall survival rates.

It is always the goal of early HCC monitoring and diagnosis to detect low-content tumor markers in the early stage of tumor progression and improve the sensitivity, specificity, and stability of detection. Because the identification means of HCC early screening markers are still limited, HCC biomarkers used for prognosis evaluation may gain more clinical value in the future [109].

#### Diagnostic technology based on imaging examination

##### Nano imaging technology combined with ultrasonography

Ultrasound is an effective, inexpensive, and non-invasive method for monitoring early liver cancer. However, ultrasound often cannot clearly show the necrotic area and tumor boundary. The requirements for clinical images in early monitoring and diagnosis are different. The former should pay more attention to diagnostic sensitivity. Minami et al*.* [110]. found that, due to the special metabolism and degradation products of HCC cells, the contrast between HCC cells and surrounding tissues is obvious. Additionally, compared with other abdominal malignant tumors, the early screening of liver cancer is less vulnerable to gas interference [111]. Therefore, it is more suitable to develop ultrasound contrast agents that can distinguish liver cancer and normal liver tissues. Advanced ultrasound imaging technology improved with nanomaterials makes the diagnosis of hepatocellular carcinoma simpler and more effective. This study reports the integration of cavitation/ultrasonic perforation technology. The unique prodrug nanobubbles (NBS) added to the tissue play an *in-situ* thermal effect, which can enhance the ability of ablation and tissue penetration and can be used for histopathological analyses. As ultrasonic contrast agents, they play an important role in the early monitoring of liver cancer. Because this nanomaterial can convert ultrasonic energy into heat, it is also used in collaborative hyperthermia. For example, NBS-GPC3-reduced graphene oxide (RGO) has been developed as an ultrasonic-assisted photothermal agent because of its suitable particle size, imaging ability, and photothermal efficiency.

Singh et al. [112] found that antioxidant poly(oxalic acid curcumin) (POC) particle bubbles can generate CO-NBS through a peroxide-induced oxalate of peroxide, which has a potential role in both enhancing ultrasonic diagnosis in early stage in HCC and improving liver function of HCCs.

##### Nanoimaging combined with CT and MRI

In recent years, the development of nanomedicine continues to promote the accuracy of CT imaging, and more and more efficient CT contrast agents have been developed. Wang et al.. [113] applied asymmetric magnetic mesoporous silica NPs in diagnosis and treatment in HCC to reveal its advantages in theranosis of HCC with suicide genes/precursor drugs guided by magnetic enhancement and imaging, as wel as magnetocaloric therapy. Subsequently, the improved gold mesoporous silica Janus nanoparticles (GSJNs), as HCC-targeted CT imaging agents, are used as an integrated platform for tumor diagnosis, radiotherapy, and chemotherapy, significantly reducing systemic toxic and side effects, rendering the multifunctional Janus nanosystem a promising platform for achieving efficient and safe HCC treatment [114]. Additionally, chemicals with different attenuation coefficients, as accurate targeted CT imaging agents for HCC, have also become promising reagents for the safe and effective clinical diagnosis and treatment of HCC in the future.

In the field of MRI imaging, gadolinium, as a traditional MRI contrast agent, has a nonspecific distribution in the body, low sensitivity to early tumors, and certain nephrotoxicity. Additionally, gadolinium has a good imaging effect in kidney and brain tissues, but is poor in the hepatobiliary system. Therefore, superparamagnetic iron oxide nanoparticles (SPIONs) were born as more efficient MRI contrast agents for the hepatobiliary system. They are magnetic particles with iron oxide nanoparticles as the core and colloidal properties. They were approved by the U.S. Food and Drug Administration (FDA) for clinical application in 1996 and are the most widely used specific liver MR contrast agent for clinical applications [115].

Nanoparticles designed by Wu et al *.*[116] can transfer extracellular vesicles to tumor-specific continuous nanocatalysts for MRI nanocatalytic therapy of hepatocellular carcinoma. Glucose consumption is catalyzed by loaded glucose oxidase, excess hydrogen peroxide is produced, and a Fenton-like and highly toxic hydroxyl radical-catalyzed reaction is produced by extracellular vesicle NPs. The uniqueness of these nanoparticles is that they show a high active targeting ability to achieve efficient tumor inhibition in vitro and in vivo. Additionally, the synthesized nanoreactor can also be used as an ideal nanocontrast agent for MRI, showing an ideal nanocatalytic imaging ability in the continuous imaging process.

#### Pathological diagnosis technology based on liver biopsies

In general, suspected liver space-occupying lesions lacking typical tumor imaging features can be diagnosed by liver biopsy cytology or histology. In the relatively indirect electromagnetic field, the ultrastructure saves staining time, widens the perspective of postoperative pathological diagnosis of liver cancer, and provides a better idea to improve the accuracy and efficiency of liver cancer diagnoses. With the development of precision medicine, in the future, tumor genome information obtained by liver biopsy will also be used as an important drug basis for molecular targeted therapy [115].

## Application status of different technologies for the treatment of liver cancer

The nanotherapy of liver cancer is mainly achieved by combining chemotherapy, radiotherapy, targeted therapy, and other types of technology. At present, nanopreparations that can be used for drug embedding include polymer nanoparticles, lipid nanoparticles, silica nanoparticles, and liposomes.

### Nanotherapy of liver cancer combined with chemotherapy

Because chemotherapy can act on areas that cannot be reached by surgery, radiotherapy, and other methods and also effectively kill metastasizing cancer cells and prevent the recurrence and metastasis of cancer. Encapsulated in various nanoparticles, chemotherapy drugs can selectively deliver therapeutic drugs to tumor sites through DDSs, improve the duration of local blood drug concentrations, and reduce systemic adverse reactions. To a certain extent, nanoparticles overcome the limitations of traditional chemotherapy and show great potential in the precise treatment of cancer. Additionally, nanotechnology can guide the release of drugs in HCC lesions by relying on the convergence of tumor-specific P-selectin and the catalysis of the tumor microenvironment, to allow chemotherapeutic drugs to better act on the solid tumor itself [117].

### Nanotherapy of liver cancer combined with radiotherapy

As liver cancer is not very sensitive to radiotherapy, radiotherapy is not used as a routine first-line treatment for it but is often used in the palliative treatment of advanced liver cancer to improve the quality of life of patients, especially for patients with bone metastasis. In recent years, the new strategy of combining new radiotherapy sensitizers and radiotherapy and chemotherapy has become a research hotspot. After their toxicity detection and accurate safety evaluations, these nanomaterials could be put into clinical applications. For example, platinum-containing surface ligands can be used to enhance X-ray-induced photodynamic therapy (X-PDT) [118], which has the advantages of high tissue penetration of X-rays and efficient generation of hydroxyl radicals by photodynamic therapy. However, the transformation of X-rays still restricts the development of X-PDT [115]. With the rapid development of nanotechnology, nano-assisted radiotherapy has achieved improvements in anti-cancer treatment targeting, patient prognosis and quality of life, which has brought more opportunities to overcome the bottleneck of radiotherapy [119].

### Nanotherapy of liver cancer combined with targeted therapy

Targeted therapy can be classified based on passive targeting, active targeting, and physicochemical targeting.

####  Passive targetin

Refers to nanobiomaterials that are phagocytized by the endothelial reticular structure of the liver tissue phagocytosis system based on an EPR effect, whereby drugs are passively enriched in the tumor area through the physiological process of phagocytosis. Common passive targets include liposomes, nanoparticles, emulsions, microspheres, etc. [120].

####  Active targetin

Refers to the combination of specific ligands connected to nanomaterials and the overexpression of characteristic receptors on the tumor cell surface to further improve the enrichment of tumors and reduce the toxic and side effects on normal tissues. If two ligands are modified to form double targets, the targeting will be further improved [121, 122]. Many studies [123–126] have shown that tumor-associated macrophages (TAMs) are abundant in the tumor microenvironment (TME), which can have an important impact on tumor metastasis, angiogenesis, and immune escape. Strategies for passive targeting to liver cannot meet the needs of current basic and clinical research, and active targeting nanocarriers based on the characteristics of the TME have been increasingly designed [127]. Compared with traditional passive targeting, active targeting has shown superior efficacy in basic research. Aptamers have a unique spatial structure and membrane penetration ability and can be used as active and passive anticancer targeted delivery drugs, to double their intracellular concentration.

####  Physicochemical targeting

Is a targeted drug delivery system that uses some physicochemical methods to allow drugs to exert their efficacy at specific sites. According to the characteristics of the TME, internal stimuli include pH level [115], redox state [128, 129], enzyme targeting [130–132], while external stimuli include light targeting [115], thermal targeting [133], magnetic targeting [134], and embolization targeting. For example, the GSH-responsive degradation nanodrug carriers are designed based on the degradability of disulfide bond to high concentration GSH in tumors by synthesizing organic–inorganic hybrid nanosilica spheres (doped disulfide bond structure) to connect liver cancer-specific ligands. Nanocarriers with GSH-responsive degradation and drug release properties were obtained, and can effectively enhance the efficacy of liver cancer chemotherapy and reduce systemic toxicity and side effects. Recently, dual responsive nanogels were developed to prevent premature drug leaching and achieve efficient intracellular drug release [135].

Because tumor immunogenic cell death (ICD) induced by traditional local treatment is usually not enough to cause systemic effects against metastasis or prevent tumor recurrence, local regional heat treatment has become the most commonly used method to induce ICD, including photothermal and magnetothermal therapies [136]. The commonly used magnetic target is composed of magnetic substances, drugs, and fluorescent agents. Drugs are used to kill cancer cells, magnetite (Fe3O4) nanoparticles are used for targeted positioning, and fluorescent agents such as rhodamine B are used for fluorescence tracing and real-time display. Studies [137, 138] have shown that through the induction of mitophagy and apoptosis, synergistic NPs can rebuild an immunosuppressive microenvironment, consequently motivating and promoting the ICD of human HCC cells. This strategy offers the prospect of significant remission in HCC patients with reduced liver function due to its high efficiency and relatively low toxicity.

Nanoknife technology, which has been approved for clinical tumor ablation since 2012, can release high-voltage pulses to tumor cells through the probe, making their cell membrane produce nano-irreversible electroporation, resulting in tumor cell apoptosis, after which the treatment area is gradually replaced by normal tissue. Nanoknife technology [139] has advantages over other ablation technologies. It does not generate heat during the operation or rely on heat and is not affected by the blood flow of adjacent large blood vessels. The cell death caused is not necrosis, but apoptosis, and can stimulate the anti-tumor immune response. The treatment time is very short, which is conducive to postoperative rehabilitation. The ablation process can be clearly displayed on ultrasound, CT, or MR, to ensure the maximum therapeutic effect, especially for solid tumors close to the hilar region, gallbladder, and bile duct.

### Nanotherapy of liver cancer combined with other types of technologies

#### Nano gas preparation

This technology uses the tumor microenvironment as an endogenous stimulus for *in-situ* responsive gas release. The controllable drug release of the internal stimulus has the advantage of not being limited by the depth of tissue penetration and can more effectively deliver the drug to the interior of the tumor. For tumor therapy, the tumor microenvironment is an ideal endogenous stimulus, considering its acidity, reducibility, and high expression of H2O2. He et al. [140] developed nano gas drugs for hydrogen peroxide responses in tumors, named MnCO@hMSN. A selective anti-tumor gas therapy was successfully achieved for the first time. The drug selectively decomposes and releases CO gas in the tumor but does not decompose and release gas in normal tissues, which greatly improves the effect of tumor treatment and reduces the toxic and side effects of drugs, setting a milestone in the development of selective anti-cancer treatments.

Tumor vascular abnormalities have an important impact on tumor progression and response to therapy, and nitric oxide (NO) can regulate angiogenesis and maintain vascular homeostasis while regulating the activities of various metabolic enzymes through nitrification and nitrosylation reactions. Considering this, Chen and Lu et al .[141] reported NanoNO, the nanoscale carrier enables sustained NO release for efficient NO delivery to hepatocellular carcinoma, demonstrating that nanoscale NO delivery can effectively reprogram tumor vasculature and tumor vasculature. The immune microenvironment can overcome resistance in anti-cancer treatment, thereby enhancing the therapeutic benefits and providing a new strategy for liver cancer treatment. In order to improve the precise targeting and synergistic therapeutic effect of NO, Ding and Huang et al. [142] further developed a glutathione (GSH)-sensitive NO donor, BPDB. Nitroanion is present in a chemokinetic (CDT) synergistic therapeutic formulation, and realized the synergistic effect of NO-CDT therapy, while also provided a prodrug (e.g., NO donor)-based design platform for new methods of precision cancer treatment.

#### Gene therapy

Gene engineering nanomedicines based on both DNA and RNA can regulate cell apoptosis, proliferation, and survival by regulating the mitochondria-mediated apoptosis pathway, cell cycle checkpoints, and the RTK survival pathway.

##### DNA-targeted therapy

CRISPR/Cas9 can play an important role in nanomedicines. CRISPR/Cas9 is a macromolecular complex containing a nuclease (Cas9) capable of cleaving a duplex of target genome sequences. A scan of the genome to assist this nuclease finds the single-guide RNA (sgRNA) of the specific sequence to be edited. When the sgRNA is linked to the Cas9 enzyme and recognizes the target DNA sequence, the Cas9 enzyme will target and cut the DNA [143]. Because this gene-editing tool can precisely change the genetic code of cells, it is expected to achieve precise diagnosis and treatment of liver diseases, although it can only play its role in the nucleus.

##### RNA interference therapy

Since the discovery of RNA interference (RNAi) in *C. elegans* by Nobel Prize winners Andrew Fire and Craig Mello in 1998 [144], its use in tumor gene therapy has been extensively studied. In 2019, researchers from the Chinese Academy of Sciences and Tufts University in the United States [145] developed a CRISPR/Cas9 system delivery technology, by encapsulating mRNA in biodegradable synthetic lipid NPs, which had significantly improved liver function. Buried mRNA encoding Cas9 is delivered to the body, and after the contents are released into the cells, the targeting and cleavage of DNA are executed with a delivery efficiency of up to 90%, representing one of the most effective delivery tools reported so far. On this basis, Xue et al. [146] from the Institute of RNA Therapeutics, MIT School of Medicine, developed a CRISPR-SONIC system that can flexibly and accurately perform oncogene knock-in in a mouse model of liver cancer, meeting the needs for developing rapid and effective cancer animal models.

In 2021, Professor Siegwart's team [147] developed an organ-selective targeting (SORT) technology. They screened and optimized a new multi-tailed ionizable phospholipids (iPhos) with strong endosome escape performance, centered on 9A1P9, iPLNPs realized organ-selective mRNA delivery and optimized CRISPR CAS gene editing system. At present, Synthego is deeply cultivating the second-generation technology CRISPROff [148], which is a specific and accurate optically controlled CRISPR gene-editing technology, and improving its GMP (drug production quality management specification) production capacity to support clinical transformation and finally achieve the goal of shortening the time required for gene editing therapy. Because CRISPR/Cas9 systems can only exert their function after reaching the nucleus, safe and effective in vivo delivery is still the biggest obstacle restricting the application of gene therapy.

**Fig. 4:**
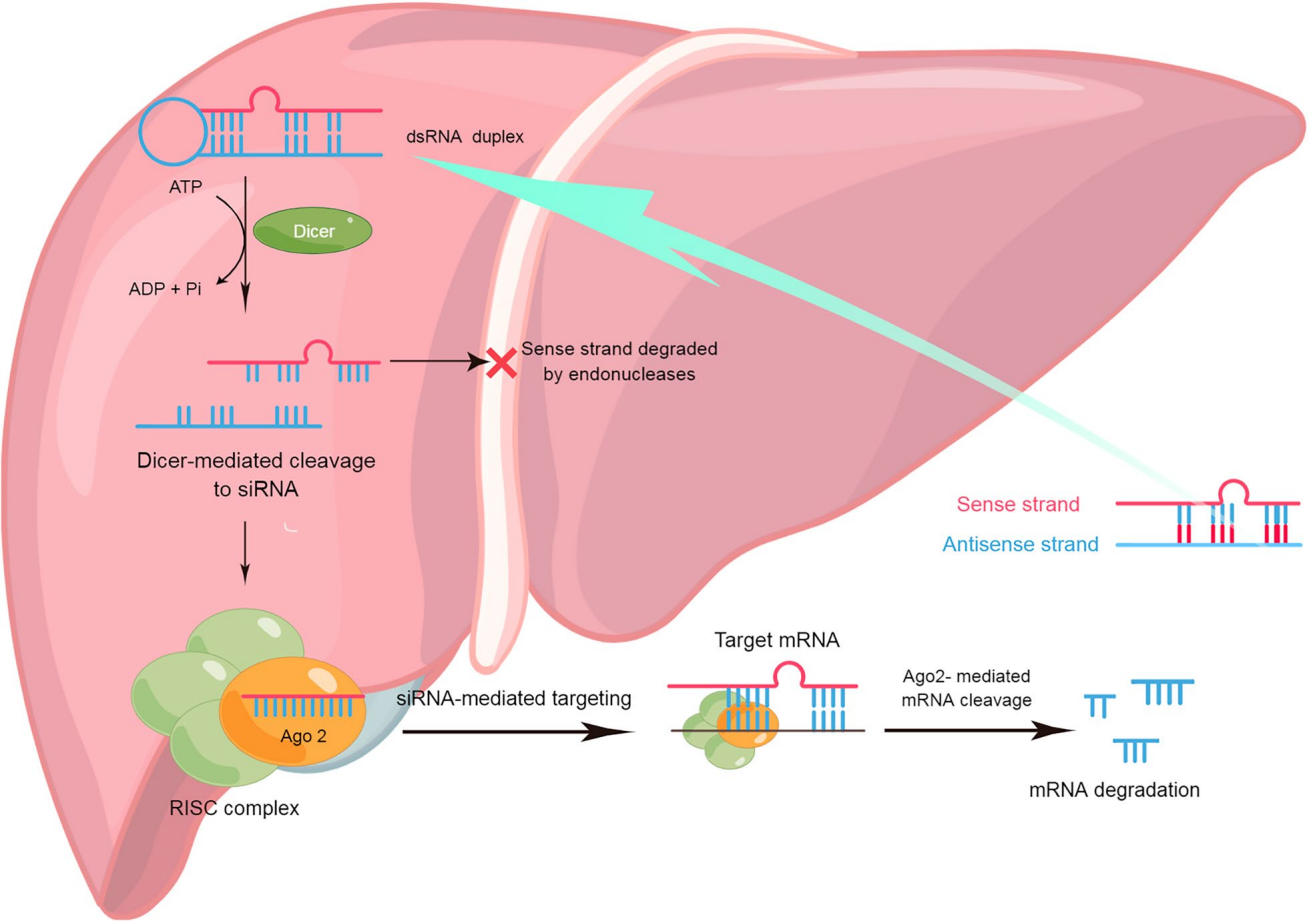
Representation of the production and mechanism of siRNA-mediated gene silencing (*ADP* adenosine diphosphate, *ATP* adenosine triphosphate, *siRNA* small interfering RNA, *mRNA* messenger RNA, *RISC* RNA-induced silencing complex**)**

Among different types of RNAi tools, small interference RNA (siRNA) comprises the core of RNAi [149]. siRNA uses a short dsRNA generated in the cell after an exogenous long dsRNA enters the cell and is cut by Dicer (of the RNA-III nuclease family). These siRNAs are separated by helicase, and their antisense strand (or guide strand) is embedded in the RNA-induced silencing complex (RISC). The sequence specifically guides the combination of RISC and complementary target mRNA, so as to effectively and selectively silence the transcription of mRNA, resulting in the downregulation of the corresponding gene’s expression (Fig. 4, By Figdraw, www.figdraw.com) [150].

**Fig. 5:**
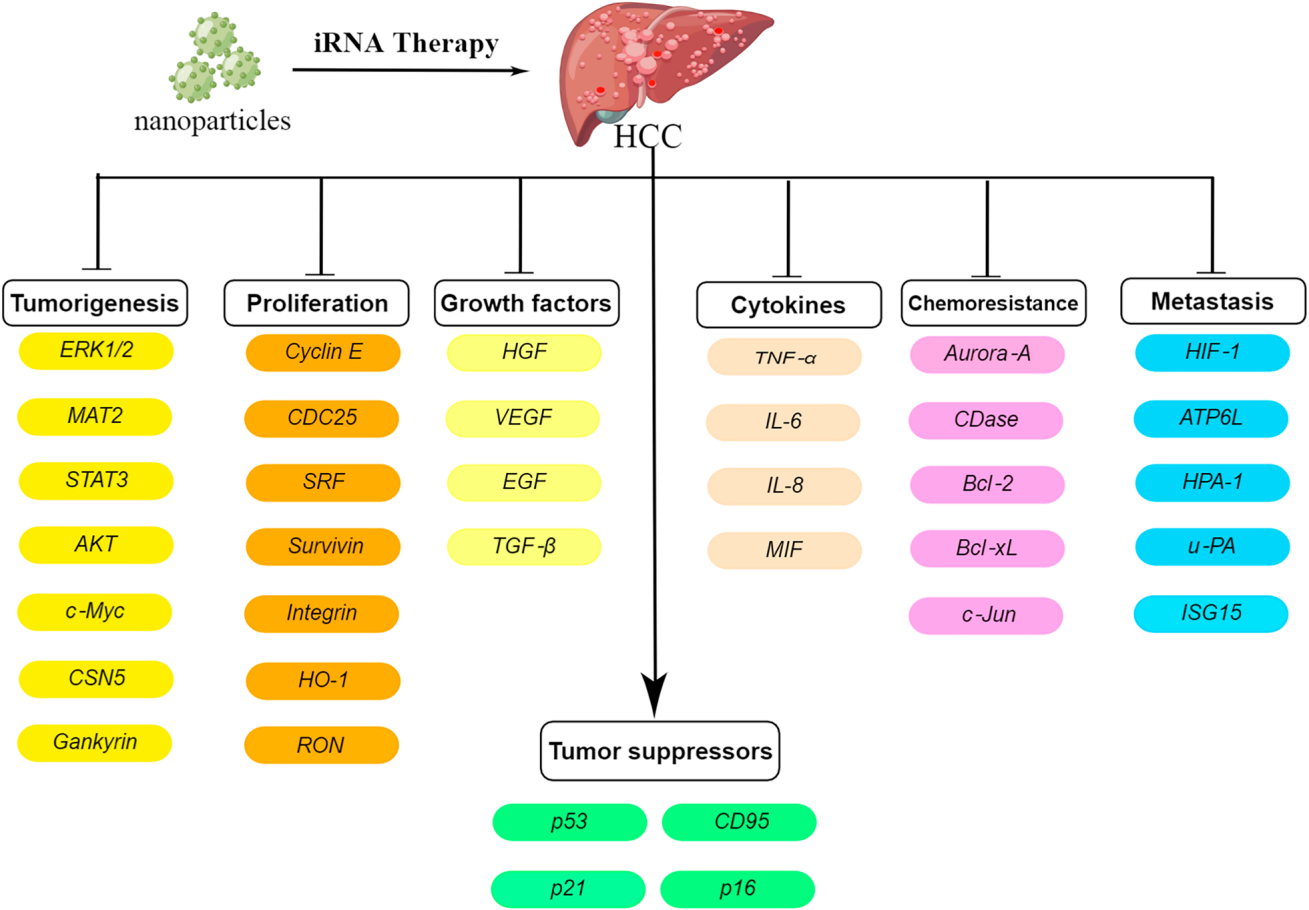
Strategies for iRNA therapy of HCC-promoting genes and upregulation of tumor suppressor genes

Up to now, the strategies of siRNA-targeted therapy of HCC mostly target (Fig. 5, By Figdraw, www.figdraw.com) liver inflammatory injury [151–154], cancer pathway [155–158], tumor occurrence and development [159–169], and tumor metastasis [170–176]. Another study [177] modified nanoparticles with azobenzene, a hypoxia-responsive substance, and applied them to tumor gene therapy, which not only improved the drug targeting effect but also effectively increased the phagocytosis efficiency of siRNA by tumor cells, so as to achieve a considerable antitumor effect.

Self-amplifying RNA (saRNA) is a small-molecule double-stranded RNA, with a chemical structure similar to that of siRNA, which has the function of specifically upregulating the expression of targeted genes. Based on this, MiNA Therapeutics [178, 179] designed a new drug MTL-CEBPA, which is made by Smarticles ® Liposome nanoparticles composed of double-stranded RNA, which is used to activate the CEBPA gene, the main regulator of liver function, returning C/EBP-α protein to normal levels to reduce the immunosuppressive effect of myeloid cells. It shows the advantages of fighting cancer immune escape and improving the activity of anticancer drugs in preclinical and clinical studies. At present, MTL-CEBPA is being implemented in the first-line targeted drug treatment of patients with advanced HCC [180]. The administration of the first phase I clinical trial patient was completed by the end of 2021. It is expected that the final results will bring the gratifying news that the objective remission rate (ORR) of patients can be improved after treatment, to further confirm that a new immune combination therapy containing MTL-CEBPA may significantly improve the standard treatment of patients with advanced HCC. Recently, Shi et al. [181] developed lipid nanoparticles composed of G0-C14, PLGA, and lipid PEG, and modified them with the targeted peptide CTCE-9908 with specificity for CXCR4 (highly expressed in liver cancer cells), then specifically delivering p53 mRNA to liver cancer cells and using it in combination with immune checkpoint blocking therapy, which can effectively induce the global reprogramming of the TME and reverse the immunosuppression of liver cancer.

Because a decrease in the transcriptional levels of tumor-suppressive miR26a and miR122 will lead to abnormal proliferation, chemoresistance, and distal migration of liver cancer cells, it is expected to become an efficient means to realize HCC gene therapy by delivering exogenous tumor-suppressive miRNA and restoring the balance of miRNA in tumor cells. However, due to the degradation of miRNA by nucleases, the construction of a stable gene vector is still the key of miRNA gene therapy. The PEG-BCPVs/miRNA treatment system constructed by Yong, Chen & Huang et al. [182]*.* in 2018 can safely and effectively transfect HCC cells with miR26a and miR122 at the same time, significantly inhibiting the expression of multiple targeted oncogenes.

RNA interference therapy is a broad field to be developed. In addition to the above technologies, it also includes antisense oligonucleotides and nucleic acid aptamers [183, 184]. It is more flexible and multifaceted than DNA-based therapy. Despite the heterogeneity of the tumor environment, the specificity of controlled RNA release, the transfection efficiency in tumor cells, the delivery strategies, the off-target effects, interferon effects, and safety of nanomedicines are still limiting the clinical translation of RNAi technology. The characteristics of high specificity, rapidity, simplicity, and efficiency of gene therapy are still widely favored by scientists. RNAi technology is expected to become a revolutionary cancer treatment method.

In 2018, Chen et al. [185] first clarified that targeted deep sequencing can overcome tumor heterogeneity, find therapeutic target, and greatly improving the success rate of xenograft tumor model (PDX) modeling from 25.5% to 42.2% in HCC patients, and establish the first international PDX database to guide clinical individualized treatment.

## Conclusions

Nanotechnology has become a new frontier discipline in medical testing and materials science. Different from traditional targeting agents or chemical carriers, the research and development of nanotechnology has always focused on combining its unique properties with drugs for the imaging and treatment of HCC, aiming to promote its precise treatment [186]. Nowadays, nanodrug carriers in the market are still mainly liposomes. Although liposomes have many advantages, they retain problems such as low encapsulation efficiency and low storage stability. Therefore, a more complex multifunctional design is needed to achieve similarity with the biological environment as much as possible, to successfully cross the biological barrier and improve the prognosis of patients after administration [187]. In recent years, new drug carrier polymer nanoparticles (PNP) have been given great attention. Compared with liposomes, they not only have the same advantages of targeting specificity and low side effects, but also have higher stability (especially protein drugs) and better slow/controlled release. At present, PIHCA polymeric nanoparticles used in the treatment of advanced liver cancer have achieved considerable curative effects in phase III clinical trials [188].

After decades of in-depth research, many preclinical studies of DDS combined with nanomaterials have been published, and some nano-drugs for HCC-targeted treatment of liver cancer have also entered the stage of clinical trials, although they have not been successfully transformed into large-scale practical clinical applications. This shows that finding out physiological hurdles in the human body and its corresponding solutions is vital in guiding the rational design of HCC targeting nanomedicine systems. we should come up with pointed molecular biological approaches and establish proper physiological criteria to study HCC specific inhibitory methods. The purpose of surface engineering of nanoparticles is to escape the phagocytosis by RES, prolong blood circulation time, and improve bioavailability. Additionally, combined with the characteristics of the liver cancer microenvironment, nanoparticles are endowed with a corresponding functional design to achieve better tumor response and targeting [189].

So far, clinical demand has driven nanomedicine and many emerging treatment methods to achieve the individualized treatment of liver cancer, but the following three problems restricting the development of nanodrugs still remain: 1. Most of the problems in the research and development of nanodrugs for liver cancer focus on the drug-loading synthesis process, and achieving the reversible combination between nanopreparations and drugs remains very important. As anti-tumor drugs themselves are constantly changing and substituting old ones, there are great differences between different drugs. Their onset mechanism and pharmacokinetic characteristics increase the difficulty of the research and development of nanodrug loading systems. 2. The measurement of separating encapsulated and unpackaged components are equally important for determining the bioequivalence of general nanodrugs. However, current methods for measuring drug release in the plasma are limited, and inevitably causing illusion from non-equilibrium conditions and processes [115]. 3. The lack of standardized biosafety evaluation in many studies could not guarantee that some agents, especially inorganic NPs with large diameters. For instance, toxicity to the liver and kidneys caused by heavy metals, harm to the walls of blood vessels caused by acids and alkalis, and hemodynamic changes caused by many blunt substances which can lead to organ failure in the human body. The evaluation system of drug loading efficiency and drug loading toxicity of NPs needs to be further optimized. The limitation of clinical translation of NPs is closely related to the preciseness, standardization, and repeatability of relevant biological experiments, requiring future research to pay more attention to its application in the treatment of solid liver cancer. It is believed that, with the efforts of many scientists, nanomedicines will eventually become an indispensable part of cancer diagnosis and treatment.

## Data Availability

Not applicable.
